# Incipient Constituents: Phonesthemes Facilitate Word Processing in English

**DOI:** 10.1162/OPMI.a.360

**Published:** 2026-06-17

**Authors:** David A. Haslett, Bo Yao, Xufeng Duan, Zhenguang G. Cai

**Affiliations:** Division of Social Science, The Hong Kong University of Science and Technology; Department of Psychology, Lancaster University; Department of Linguistics and Modern Languages, The Chinese University of Hong Kong; Brain and Mind Institute, The Chinese University of Hong Kong

**Keywords:** form–meaning systematicity, morphology, language evolution, iconicity, ERP, EEG, phonesthemes

## Abstract

Phonesthemes are sound clusters that recur in words with related meanings. For example, *glow*, *gleam*, and *glitter* begin with the phonestheme /gl/. Experiments with pseudowords provide evidence that phonesthemes affect how people retrieve and represent meanings, which suggests that phonesthemes capture language evolution in action, namely the emergence of compositionality. However, there is little evidence that phonesthemes affect how people process familiar words with established meanings. We therefore investigated whether phonesthemes facilitate visual word recognition and affect semantic representations in English. In two lexical decision experiments, people recognized words that contain phonesthemes faster and more accurately than control words, and phonesthemes amplified N170 ERPs; and in four lexical decision megastudies, phonesthemes predicted faster visual word recognition (but not faster auditory recognition). In two semantic relatedness experiments, we found only limited evidence that phonesthemes affect interpretation: Decisions were slower and less accurate for words containing incongruent phonesthemes (e.g., /gl/ misled people about the meaning of *glove*), but only in the online experiment, with a marginal effect of accuracy in the lab-based experiment and an amplified N400 ERP in frontal sites (but not the expected centroparietal sites). Our findings demonstrate that people process words which contain phonesthemes differently than they process controls, consistent with the hypothesis that phonesthemes inhabit a middle ground between morphemes and meaningless strings of letters, but our findings also demonstrate that, unsurprisingly, these quasi-meaningful constituents have only small effects in how people represent the meanings of familiar words. We discuss implications for morphology, language evolution, and natural language processing.

“Usually a blend is a nova in the verbal heavens that flashes and then fades. But now and then one continues to glow. … Thus constellations of words have been born.” Dwight Bolinger, *The Life and Death of Words (1953)*

## INTRODUCTION

Phonesthemes are sound clusters that recur in words with related meanings, such as /gl/ in *glazed*, *glossy*, and *glisten* or /dr/ in *drip*, *drizzle*, and *drool* (Bolinger, 1940; Firth, 1964; for a review, see Hutchins, 1998). As an intermediate stage between morphemes and meaningless chunks of words, phonesthemes seem to capture the emergence of compositionality. However, despite many corpus studies and experiments with pseudowords, there is little evidence that phonesthemes affect how people process established lexical items (Bergen, 2004, being a notable counterexample). We therefore investigated whether phonesthemes facilitate visual word recognition and guide word meaning access in English.

### Phonesthemes, Morphemes, Form–Meaning Systematicity, and Iconicity

Phonesthemes are a classic example of form–meaning systematicity, the phenomenon whereby formally related words are also semantically related (for reviews, see Dingemanse et al., 2015, and Haslett & Cai, 2024). Phonesthemes are defined as sub-morphemic units, since morphologically complex words decompose to meaningful and productive constituents, while phonesthemic words may be simplex. For example, *quickly* and *hasten* can be rearranged to form *quicken* and *hastily*, whereas in *glimmer* and *drizzle*, /gl/ and /dr/ cannot be extracted without rendering the remaining constituents meaningless (Kwon & Round, 2015).

However, it is often ambiguous whether a word is morphologically complex and whether a morpheme is truly productive. For example, MorphoLex (Sánchez-Gutiérrez et al., 2018) annotates *iodine* and *chlorine* as each decomposing to /ine/, yet it considers *saline* and *morphine* to be simplex, implying that, in those cases, /ine/ is a vestige of erstwhile productivity (cf. Hopper, 1994). In contrast, many phonesthemes seem to be meaningful and productive: Phonesthemes are overrepresented in blend words, such as /sl/ and /fl/ in *slosh* and *flurry* (Smith, 2014); people tend to impose the meanings of phonesthemes on pseudowords, such as inferring that *glip* refers to something shiny (Abelin, 1999; Hutchins, 1998; Kwon, 2017; Magnus, 2000); and phonesthemes have motivated semantic change in some English words, such as *flourish* shifting towards *flaunt* and *flatter* (Smith, 2016). Driving home this parallel to morphemes, Bergen (2004) provides evidence from priming that phonesthemes facilitate visual word recognition. For example, people recognize *glisten* faster after exposure to *glow* than *shine* or *glove* (though note that Bergen used a between-items design), which suggests that phonesthemes are psychologically real constituents and that people automatically decompose words to phonesthemes. The same is true of morphemes. For example, *depart* speeds up recognition of *department* more than semantic relatedness, orthographic overlap, or their additive effects explain (e.g., Feldman, 2000; Longtin et al., 2003; Rastle et al., 2000, 2004). Bolinger (1950) and Kwon and Round (2015) therefore conclude that phonesthemes blur the line between morphemes and meaningless chunks of words.

In this way, phonesthemes capture language evolution in action. Over the course of generations, languages increase compositionality, as supported by observations of Nicaraguan Sign Language (Senghas et al., 2004) and experiments with toy languages (Clay et al., 2014; Kirby, 2000; Kirby et al., 2022): People decompose holistic signs into serial segments, so complex meanings come to be represented by complex forms with reusable constituents. In spoken languages, quasi-meaningful and quasi-productive phonesthemes attest to an intermediate stage in the emergence of reusable constituents. Zingler (2017) provides corpus evidence that over the past 500 years, the number of English words that begin with /gl/, /fl/, and /sn/ has increased, but the proportion of phonesthemic versus arbitrary meanings (e.g., *glow* versus *glove*) has changed little. If sub-morphemic constituents were fossilized and infertile, arbitrary meanings should have instead diluted phonesthemic meanings (as has happened with /sl/), so Zingler concludes that the semantic influence of /gl/, /fl/, and /sn/ persists in word formation.

The meaningfulness and productivity of phonesthemes is consistent with distributed theories of morphology, which argue that, as with phonesthemes, the psychological reality of morphemes stems from their tendency to recur in semantically related words (e.g., Baayen et al., 2011; Plaut & Gonnerman, 2000; for reviews and important qualifications about the status of morphemes in this paradigm, see Amenta & Crepaldi, 2012, and Stevens & Plaut, 2022). These theories are backed by computational models like fastText, which can represent the meanings of pseudowords based on the linguistic contexts where subword strings occur (Bojanowski et al., 2017). For example, the phonestheme /ump/ occurs in sentences that contain *plump, bump*, or *rump*, which informs fastText’s representation of the pseudoword *vump*. Gatti et al. (2023) found that people are slower and less accurate to identify, e.g., *vump* as a pseudoword when primed by *blubber*, which is semantically related to words such as *plump* (see data from Hutchison et al., 2013). This supports the view that human word processing is sensitive to form–meaning systematicity, beyond morphology strictly construed (see also Cassani et al., 2020; De Varda et al., 2025; and Hendrix & Sun, 2021).

One limitation of this line of research is a reliance on pseudowords, since pseudoword processing may not generalize to word processing. The phonesthemes /ump/ and /gl/ affect interpretations of pseudowords such as *vump* and *glip*, but it is not clear whether they affect representations of established English words such as *trump* and *glove*. Corpus studies confirm that phonesthemes exist in English and other languages (Abramova et al., 2013; Bergen, 2004; Blust, 2003; Drellishak, 2006; Fordyce, 1988; Gutiérrez et al., 2016; Liu et al., 2018; Otis & Sagi, 2008), but as far as we know, Bergen (2004) is the only study to directly investigate the impact of phonesthemes in word processing—that is, using established lexical items in a familiar language, excluding the processing of pseudowords and foreign words. Related work has shown that form–meaning systematicity facilitates visual word recognition in English, Italian, and Malay (Amenta et al., 2017; Marelli & Amenta, 2018; Marelli et al., 2015; Maziyah Mohamed & Jared, 2025; Siegelman et al., 2022), but those studies provide evidence about the psychological salience of degrees of systematicity (basically, the semantic similarity of phonological neighbours) and therefore gloss over the role of phonesthemes in particular. Phonesthemes support distributed theories of morphology by bridging the gap between form–meaning systematicity and the psychological reality of fuzzy, morpheme-like constituents that those theories seek to explain.

Phonesthemes are often discussed in terms of iconicity, which Winter et al. (2026) define as “a sense of resemblance between at least some aspect of [a word’s] form and at least some aspect of its meaning” (p. 3). For example, *kerplop* and *fastidiousness* are iconic because they sort of sound like what they mean. Although iconicity and form–meaning systematicity are in principle orthogonal (Nielsen, 2016), they often co-occur, so systematicity is not *the* essential feature of phonesthemes. For example, the phonesthemes /op/ and /sn/ occur in iconic words, such as *plop* and *flop* or *sniff* and *snore*. On the other hand, classic effects of iconicity occur cross-linguistically (e.g., Ćwiek et al., 2022), whereas the influence of phonesthemes is often language specific (e.g., Fordyce, 1988; Kwon, 2017). Indeed, /gl/ is one of the most common examples of an English phonestheme, yet /gl/ does not seem to have any intrinsic feature that mimics meanings such as *glow* or *glimmer* (Bolinger, 1950, 1953).

The ambiguous iconicity of phonesthemes complicates claims about word processing. Iconic words are acquired early in life and are over-represented in child-directed speech (e.g., Laing 2019; Perry et al., 2015; Vinson et al., 2008), and they seem to facilitate word learning (e.g., Caselli & Pyers, 2017; Imai et al., 2008; Thompson et al., 2012), but the effects of iconicity in adult word processing are mixed. On the one hand, Meteyard et al. (2015) found that iconicity facilitated word recognition in people with aphasia; Sidhu et al. (2020) found that iconicity facilitated recognition in two lexical decision experiments; and Lockwood and Tuomainen (2015), Peeters (2016), and Vigliocco et al. (2020) found electrophysiological differences when people read iconic versus arbitrary words. On the other hand, Peeters (2016) found no difference in word recognition response time, and De Varda et al. (2025) and Sidhu et al. (2020) found no difference in response time when analyzing lexical decision megastudies. Sidhu et al. (2020) further found that the advantage for iconic words increased over the course of their experiments, consistent with exposure to iconic words leading to task-specific effects. So in this study, we must bear iconicity in mind when investigating whether phonesthemes affect word processing, and we must consider whether any such effects are task specific and/or due to list composition (e.g., an over-representation of iconic words in stimuli).

### The Present Study

Corpus studies and experiments with pseudowords provide evidence that phonesthemes are quasi-morphological constituents, affecting how people retrieve and represent meanings. We aim to provide stronger support for these claims with evidence from English word processing. Specifically, we hypothesize that phonesthemes will facilitate visual word recognition and affect interpretation.

#### Lexical Decisions.

Following Sidhu et al. (2020), we first conducted two lexical decision experiments with visually degraded words, in which participants decide whether a string of letters form an English word (e.g., *glint* is a word; *glink* is not). Whereas Sidhu et al. masked whole words (i.e., alternated letter strings and random symbols), we alternated presentation of the first and second half of a string. Phonesthemes tend to be either onsets or rimes, so this method investigated whether exposure to isolated phonesthemes, in half of a word, facilitates lexical access. This would be consistent with the theory that morphology emerges from regularities in form–meaning mapping (e.g., Baayen et al., 2011) and with evidence that such regularities facilitate visual word recognition (e.g., Marelli et al., 2015).

Along with behavioural measures (speed and accuracy), we recorded EEG amplitude. Following Vigliocco et al. (2020), we investigated ERPs in early, middle, and late time windows. Compared to pseudowords or unfamiliar symbols, words elicit larger N170 ERP components (e.g., Maurer et al., 2005), so we expect clearly presented words to elicit larger N170s than visually degraded words. Crucially, if phonesthemes mitigate the processing costs of visual degradation, they should increase N170 amplitude compared to visually degraded control words. Visual word recognition also typically elicits an N400 ERP, with greater amplitude for pseudowords than words (cf. Kutas & Federmeier, 2011), so we expect degraded words to elicit larger N400s than clearly presented words because they are harder to identify as words. However, lexical decisions require only shallow semantic processing, so we are not confident that phonesthemes will attenuate the predicted N400 effect. Finally, we investigated whether phonesthemes and other words differ in postlexical processing. For example, greater morphosyntactic complexity leads to greater P600 amplitude (e.g., Felser et al., 2003; Kaan et al., 2000; Phillips et al., 2005; cf. Brouwer et al., 2012), so the quasi-morphological complexity of phonesthemes could lead to larger P600s than controls. However, morphological complexity in lieu of grammatical errors less reliably enhances P600s (e.g., Gouvea et al., 2010; Mehravari et al., 2015), so lexical decisions about single words might not elicit such an effect.

Along with the online and lab-based experiments, we conducted a re-analysis of four lexical decision megastudies: the English Lexicon Project (Balota et al., 2007), the British Lexicon Project (Keuleers et al., 2012), the Auditory English Lexicon Project (Goh et al., 2020), and the Massive Auditory Lexical Decision database (Tucker et al., 2019). These megastudies comprise thousands of words, so any effect of phonesthemes or iconicity on word recognition is unlikely to stem from list composition (cf. Sidhu et al., 2020).

#### Semantic Relatedness Decisions.

Much research into morphology and form–meaning systematicity relies on visual word recognition (e.g., Marelli et al., 2015; Rastle et al., 2004). However, studies with pseudowords (e.g., Hutchins, 1998) and a corpus analysis (Smith, 2016) imply that phonesthemes affect how people interpret words. This would provide stronger evidence that phonesthemes hold a quasi-morphological status, reflected not only in decomposition but also reanalysis. We therefore conducted two semantic relatedness experiments with English words (i.e., asking whether pairs of words have similar meanings). Vigliocco et al. (2020) report larger N400 ERP components for onomatopoeic target words than controls, but only in semantic relatedness decisions about unrelated cues and targets, so we focused on targets which are unrelated to cues (e.g., *glove* to *shine*). The critical targets contain misleading phonesthemes (e.g., unlike many /gl/ words, *glove* is unrelated to light), which tests whether phonesthemes influence how people represent the meanings of familiar English words, as for pseudowords (e.g., Hutchins, 1998). Note that unlike Bergen (2004), Rastle et al. (2004), and others, we are not investigating masked priming or subliminally presented words, nor do these cues and targets share morphemes, pseudo-morphemes, or phonesthemes. Following Haslett and Cai (2023), we expect these misleading word forms to result in slower and less accurate responses. Semantic relatedness decisions require deeper semantic processing than lexical decisions, and semantic incongruence reliably produces N400 effects, so we expect words which contain misleading phonesthemes to elicit larger N400s than controls. For example, N400 effects are elicited by unrelated pairs of words (e.g., Brown & Hagoort, 1993; Franklin et al., 2007; Holcomb, 1993; Kutas & Hillyard, 1989), words that are incongruent with sentences (e.g., Kutas & Hillyard, 1983), sentences that are incongruent with speakers (e.g., Van Berkum et al., 2008), and words that are incongruent with pictures (e.g., Friedrich & Friederici, 2004). We hypothesize that misleading phonesthemes, such as /gl/ in *glove*, will produce this electrophysiological hallmark of semantic incongruence, too. Furthermore, if P600s index the challenge of integrating constituents into complex words, and if phonesthemes are processed as quasi-morphological constituents, then words which contain misleading phonesthemes should elicit larger P600s than controls. Indeed, the incongruence of /gl/ to the meaning of *glove*, for instance, should increase integration costs relative to /gl/ in *glow*, so we have a better chance of observing a significant difference in P600 amplitude in the semantic relatedness experiment than in the lexical decision experiment.

## LEXICAL DECISIONS ABOUT VISUALLY DEGRADED WORDS

To investigate whether phonesthemes facilitate word recognition, we conducted two lexical decision experiments. The first was conducted online, and the second was conducted in a lab while measuring EEG amplitude. They have a design of 2 (word type: phonesthemic or control, manipulated within participants and between items) x 2 (presentation: degraded or clear, manipulated within participants and within items). All data and scripts are available at osf.io/mhv4b.

### Methods

#### Participants.

For the online experiment, we recruited 64 native English speakers from Prolific (37 female, 27 male; mean age = 33.9, range = 18–50). They were paid £1 for a 5-minute task. For the lab-based experiment, we recruited 36 student participants from the University of Manchester community. They were paid £20 for a 2-hour session.

#### Materials.

For the online experiment, we designed 216 items, 108 containing phonesthemes analyzed by Otis and Sagi (2008; e.g., *glitter*) and 108 meaning-matched controls (e.g., *sparkle*), along with 216 non-word fillers that share onsets or rimes with target words (e.g., *glixen* and *spaxen*). To keep the online experiment short, the items were split into four lists, each with 27 phonesthemic targets, 27 controls, and 54 fillers. Meaning-matched items (e.g., *glitter* and *sparkle*) and form-matched fillers (*glitter* and *glaxen*) were assigned to different lists. For the lab-based experiment, each participant saw all items, so we reduced the stimuli to 168 items (84 per condition) and 168 fillers. The items were assigned to four blocks, with meaning- and form-matched items / fillers in different blocks.

Table 1 summarizes controls and phonesthemic items on six lexical variables: orthographic length, word frequency (Brysbaert & New, 2009), bigram frequency (Norvig, 2009), semantic neighbourhood density (Reilly & Desai, 2017), concreteness rating (Brysbaert et al., 2014), and iconicity rating (Winter et al., 2024). As described on OSF, we computed mean bigram frequency using Google’s trillion word corpus. Semantic neighbourhood density refers to the average distance between a word and its 25 nearest taxonomic neighbours (e.g., *cow* and *bull*), which are the words most likely to cause semantic competition and impede lexical decisions (Reilly & Desai, 2017).

**Table 1.** Mean (*SD*) lexical variables, for each of the two word types in four experiments

	Lexical, in lab		Lexical, online		Semantic, in lab		Semantic, online	
	Control	Phon.	Control	Phon.	Control	Mislead.	Control	Mislead.
Length	4.8 (0.9)	4.9 (0.8)	5.1 (1.3)	5.2 (1.0)	4.8 (1.1)	5.0 (1.2)	5.1 (1.2)	5.0 (1.3)
Word freq.	3.3 (0.6)	3.3 (0.6)	3.4 (0.6)	3.3 (0.6)	3.6 (0.8)	3.5 (0.8)	3.6 (0.7)	3.5 (0.8)
Bigram (B)	30 (13)	28 (13)	31 (14)	29 (13)	**35 (17)**	**30 (13)**	**35 (16)**	**29 (13)**
Sem. dens.	80 (52)	75 (47)	**94 (74)**	**74 (46)**	**106 (71)**	**88 (61)**	100 (63)	94 (68)
Concrete.	3.9 (0.7)	3.9 (0.6)	3.8 (0.7)	3.9 (0.6)	3.9 (1.0)	3.9 (0.9)	3.9 (1.0)	3.9 (0.9)
Iconicity	**4.7 (1.0)**	**5.3 (0.8)**	**4.7 (1.0)**	**5.3 (0.7)**	**3.9 (0.9)**	**4.1 (0.8)**	**3.8 (0.9)**	**4.2 (0.8)**
word2vec					.10 (.08)	.10 (.08)	.08 (.07)	.08 (.07)
Orth. sim.					**.12 (.12)**	**.14 (.13)**	**.11 (.12)**	**.13 (.13)**

*Note*. **Bold** indicates *p*-values < .2 in paired *t*-tests (i.e., those which are not far from significant). “Length” refers to letters per word. “Word freq.” refers to word frequency (Brysbaert & New, 2009). “Bigram (B)” refers to mean bigram frequency in billions (Norvig, 2009). “Sem. dens.” refers to semantic neighbourhood density (Reilly & Desai, 2017). “Concrete.” refers to concreteness ratings (Brysbaert et al., 2014). “Iconicity” refers to iconicity ratings (Winter et al., 2024). As explained in Semantic Relatedness Decisions About Misleading Word Forms, “word2vec” refers to the cosine similarity of word embeddings for cues and targets (Mikolov et al., 2013), and “Orth. sim.” refers to inverted and normalized orthographic edit distance between cues and targets (Levenshtein, 1966; Van der Loo, 2014).

Concreteness ratings subjectively evaluate the extent to which a word can be experienced directly through the senses, on a scale of 1 to 5. Of the 384 items in the lexical decision experiments, seven lack concreteness ratings, and of the 452 targets in the semantic relatedness experiments (Semantic Relatedness Decisions About Misleading Word Forms), 14 lack concreteness ratings. We therefore elicited supplementary concreteness ratings from GPT-5 (OpenAI, 2025), since large language models have been shown to generate human-like ratings (e.g., Martínez et al., 2024; Trott, 2024). As described in full on OSF, we presented GPT-5 with examples ratings then asked it to rate the 21 items with missing values and to rate 40 test items which have human ratings. We repeated this process 10 times, using the default temperature of 1, and calculated the mean rating for the 21 items with missing values. On the 400 test items, GPT-5 concreteness ratings have a strong correlation with human ratings from Brysbaert et al. (2014) (*r* = .94), so we are confident in the simulated ratings for the 21 items with missing values.

Iconicity ratings subjectively evaluate the extent to which a word sounds like what it means, on a scale of 1 to 7. Of the 384 items in the lexical decision experiments, 18 lack iconicity ratings, and of the 452 targets in the semantic relatedness experiments, 41 lack iconicity ratings. As with concreteness, we elicited supplementary ratings from GPT-5. On the 400 test items, GPT-5 iconicity ratings have a strong correlation with human ratings from Winter et al. (2024): *r* = .84.

After supplementing concreteness and iconicity ratings using GPT-5, all words have observations for all lexical variables. In paired *t*-tests comparing phonesthemic items to controls, they have *p*-values greater than .2 for all variables except semantic neighbourhood density in the online experiment (*p* = .011) and iconicity in both experiments (both *p*s < .001). The difference in iconicity ratings is unsurprising, and as discussed above, we do not doubt that iconicity plays a role in how people process phonesthemes. Still, to control for such differences between conditions, we regressed RT, accuracy, and EEG amplitude on these six predictors and then used the residuals as our dependent variable, as described in Data Analysis

#### Procedure.

**Figure 1.**
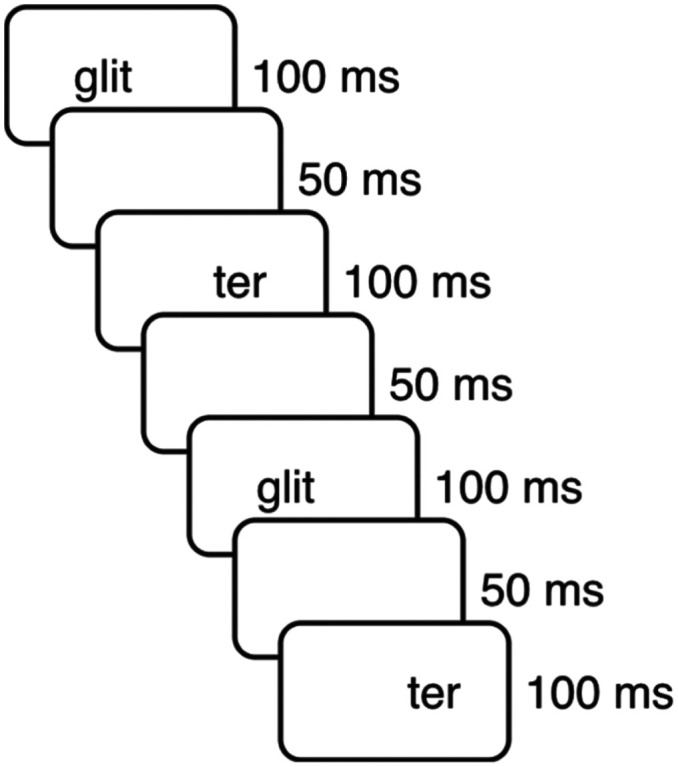
Example item from the lexical decision experiments in the degraded condition. *Note*: In the clear condition, the whole string appears for 550 ms.

The online experiment was implemented on Gorilla.sc (Anwyl-Irvine et al., 2020). The lab-based experiment was implemented on OpenSesame (Mathôt et al., 2012). For each experiment, a string of letters appeared on a computer screen, and participants indicated whether the string formed a word by pressing A or L (with the key that indicates words versus non-words counterbalanced across participants). In the degraded condition, the string flickered on the screen, alternating between the first and second half of the string, twice, with 50 ms between each appearance: first half (100 ms), blank screen (50 ms), second half (100 ms), blank screen (50 ms), first half (100 ms), blank screen (50 ms), second half (100 ms). See Figure 1. In the clear condition, a string appeared on the screen for 550 ms (i.e., the same total time). Participants had up to 2,000 ms from the first appearance of a string to make a lexical decision.

#### EEG Recording and Preprocessing.

For the lab-based experiment, we recorded EEG and EOG data with an analogue passband of 0.16–100 Hz, digitized at a sampling rate of 512 Hz, using a 64-channel Biosemi Active-Two system. The 64 scalp electrodes were mounted in an elastic electrode cap using the international 10/20 system. External electrodes were placed above and below the right eye to measure vertical ocular activity (VEOG) and were placed next to the outer canthi of the eyes to record horizontal ocular activity (HEOG). Electrode offset values were kept between −25 mV and 25 mV.

We analyzed the EEG data in Matlab, using EEGlab and fieldtrip (Delorme & Makeig, 2004; Oostenveld et al., 2011; The Mathworks Inc., 2022). Raw EEG data were re-referenced to the average signal across all EEG channels, high-pass filtered at 0.5 Hz cutoff frequency, and downsampled to 100 Hz. We interpolated bad channels using the pop_clean_rawdata() function with the default criteria (5.7 channels were interpolated on average). After interpolating bad channels, we re-referenced the EEG data to the average reference again. Using the ‘runica’ algorithm with the default options, we ran ICA including both the EEG and EOG channels and produced components using appropriate data ranks (66 minus the number of interpolated channels minus 1). Using the pop_iclabel() function (Pion-Tonachini et al., 2019), artifact components were automatically identified if they are classified as < 5% “brain” and > 80% “eye”, “muscle”, “heart”, or “channel noise” artefacts. All components were visually inspected to check for false positive classifications. On average, around 20 independent components were labelled as artefactual and removed. Finally, the data was epoched from −200 to 1,000 ms and corrected using −200 to 0 ms as the baseline, time-locked to the onset of the target word.

#### Data Analysis.

For both lexical decision experiments, we measured response time (RT) and accuracy. We excluded participants who responded correctly on less than two-thirds of trials. For RT analyses, we included only correct trials and, following Sidhu et al. (2020), excluded trials faster than 200 ms or more than 2.5 *SD* above the mean RT by subject. Using the lmerTest R package (Bates et al., 2015; Kuznetsova et al., 2017; R Core Team, 2024), we conducted linear mixed effects modelling, using forward model comparison with an alpha of .2 to determine the maximal random effect structure justified by the data (Barr et al., 2013). To control for potential confounds, we regressed RT and accuracy on the six lexical variables summarized in Table 1, and we used the residuals as our dependent variables. As we show in the online supplement, the results are almost identical when using residuals versus raw RT and accuracy, since the conditions are well matched. We sum-coded presentation (clear = −0.5, degraded = 0.5, then mean-centred) and word type (control = −0.5, phonestheme = 0.5, then mean-centred) to examine the main effects of the two variables and their interaction.

For the lab-based experiment, we measured differences in EEG amplitude during the N170, N400, and P600 windows. The N170 is a negative-going ERP component that peaks approximately 170 ms after stimulus onset over occipitotemporal sites and occurs during visual word processing (Bentin et al., 1996; Maurer et al., 2005). Because N170 effects vary in latency (e.g., Rossion et al., 2003), we measured amplitude in the P7, PO7, P8, PO8 electrodes between 150 and 250 ms, then identified the peak amplitude averaged across all conditions and investigated the 50 ms window around that peak. Because linguistic N170 effects are more pronounced in the left hemisphere (e.g., Maurer et al., 2008), we included left versus right electrodes as a sum-coded factor (P7, PO7 = 0.5; P8, PO8 = −0.5). Following Maurer et al. (2005), we excluded electrode amplitudes greater than 3 *SD* above the mean by subject.

The N400 is a negative-going ERP component that peaks approximately 400 ms after stimulus onset over centroparietal sites and is sensitive to a variety of semantic stimuli (Kutas & Federmeier, 2011; Kutas & Hillyard, 1980). To investigate the N400, we averaged amplitude in the centroparietal electrodes (C, CP, and P; 1, 2, 3, 4, and z) between 300 and 500 ms. The P600 is a long-lasting positive-going ERP component that peaks between 500 and 900 ms after stimulus onset. To investigate the P600, we measured amplitude across the same electrodes as the N400, averaged in the 200 ms window around the peak positivity in the 500–900 ms window. For both N400 and P600 ERPs, we again excluded electrode amplitudes greater than 3 *SD* above the mean by subject. As with the behavioural data, we regressed trial-level data on the six lexical variables and then regressed the residuals on the interaction of word type and degradedness in mixed effects models.

### Behavioural Results

**Figure 2.**
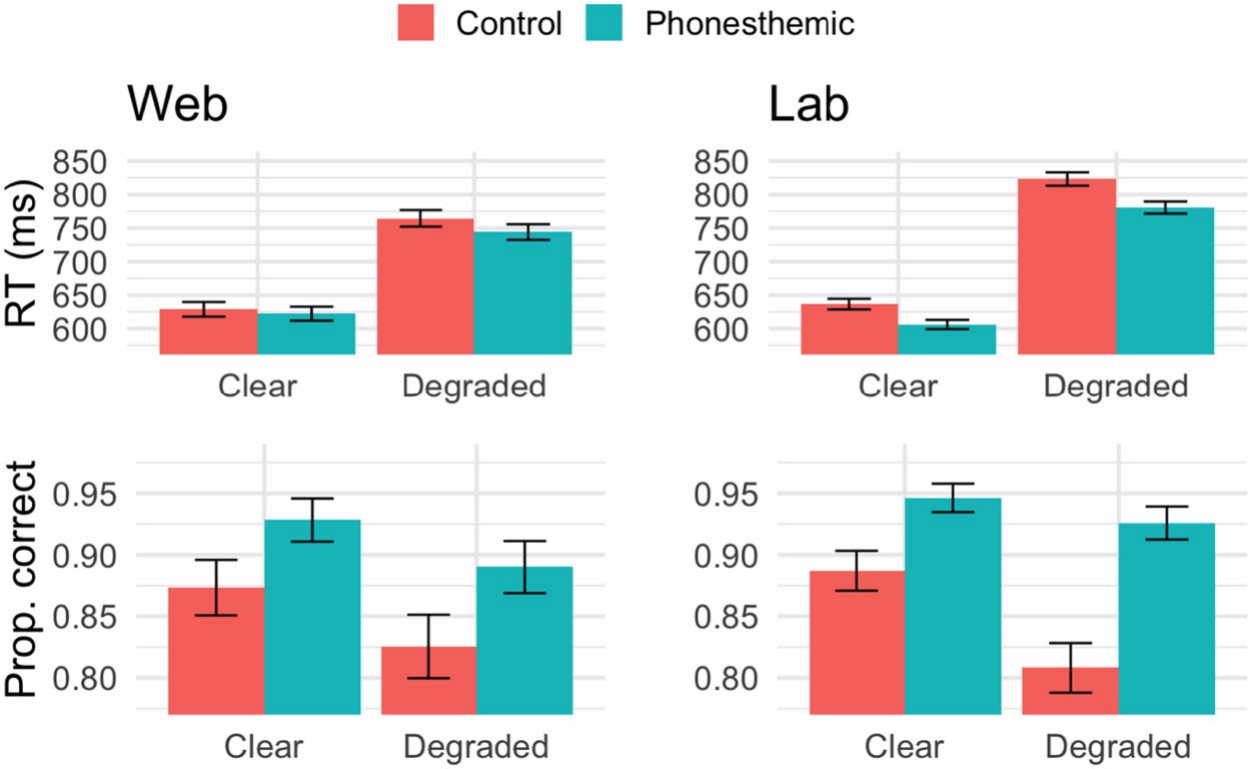
Response time and accuracy in the lexical decision experiments. *Note*: Error bars indicate 95% CIs.

For the online experiment, we excluded two out of 64 participants for low accuracy, and we excluded 70 of the 2,944 remaining trials (2.4%) for outlying RT values. For the lab-based experiment, we excluded one out of 36 participants for low accuracy, and we excluded 125 of the 5,244 remaining trials (2.4%) for outlying RT values. As shown in Tables 2 and 3 and Figure 2, we observed significant main effects of presentation on RT and accuracy in both experiments: Participants were slower and less accurate at recognizing strings as words when the strings flickered. More importantly, we also observed significant main effects of word type on RT and accuracy in both experiments: Participants were faster and more accurate when words contained phonesthemes. We expected to observe the impact of phonesthemes as interactions instead of main effects, but the interaction of word type with presentation reached significance only in the lab-based experiment, such that phonesthemes increased accuracy when words were degraded. The main effects of word type support the hypothesis that phonesthemes facilitate visual word recognition.

**Table 2.** Mean (*SD*) response time and accuracy for lexical decisions

	RT (ms)		Accuracy	
Presentation, word type	Web	Lab	Web	Lab
Clear, control	629 (150)	637 (147)	.87 (.33)	.89 (.32)
Clear, phonestheme	622 (147)	606 (131)	.93 (.26)	.95 (.23)
Degraded, control	764 (162)	823 (172)	.83 (.38)	.81 (.39)
Degraded, phonestheme	744 (160)	781 (167)	.89 (.31)	.93 (.26)

**Table 3.** Results of mixed effects regression models for lexical decisions

	Web-based RT			Lab-based RT		
	*B*	*SE*	*p*	*B*	*SE*	*p*
Word type	−17.7	5.4	.0011	−26.5	6.1	3e−5
Presentation	127.2	6.9	< 2e−16	182.3	12.5	< 2e−16
Word: Pres.	−15.3	8.5	.0714	−12.6	7.8	.1070
	Web-based accuracy			Lab-based accuracy		
	*B*	*SE*	*p*	*B*	*SE*	*p*
Word type	0.14	0.04	.0018	0.14	0.04	.0017
Presentation	−0.11	0.03	.0014	−0.13	0.03	3e−5
Word: Pres.	0.03	0.05	.5732	0.17	0.04	.0001
				N170 ERP		
				*B*	*SE*	*p*
Word type				−0.38	0.17	.0286
Presentation				−0.08	0.11	.4974
Hemisphere				−0.74	0.24	.0044
Word type: Presentation				−0.69	0.23	.0026
Word type: Hemisphere				0.12	0.23	.5925
Presentation: Hemisphere				−0.64	0.23	.0046
Word type: Presentation: Hemisphere				−0.61	0.46	.1807
				N400 ERP		
				*B*	*SE*	*p*
Word type				0.07	0.08	.3890
Presentation				−0.36	0.08	2e−6
Word type: Presentation				−0.07	0.15	.6320
				P600 ERP		
				*B*	*SE*	*p*
Word type				0.20	0.08	.0090
Presentation				−0.73	0.10	7e−9
Word type: Presentation				0.31	0.15	.0375
				N400, whole brain		
				*B*	*SE*	*p*
Word type				−0.02	0.04	.6540
Presentation				−0.02	0.04	.6730
Anteriority				−1.23	0.04	< 2e−16
Word type: Presentation				−0.02	0.08	.7780
Word type: Anteriority				−0.04	0.08	.6020
Presentation: Anteriority				1.01	0.08	< 2e−16
Word type: Presentation: Anteriority				0.23	0.17	.1780

*Note*. The dependent variables are the residuals of RT/accuracy/ EEG amplitude after being regressed on six lexical variables: Word frequency, orthographic length, bigram frequency, semantic neighbourhood density, concreteness rating, and iconicity rating. All predictors are sum coded and mean centred, with phonesthemes, degradedness, and left /anterior sites as the positive values.

### ERP Results

#### N170 Effect.

**Figure 3.**
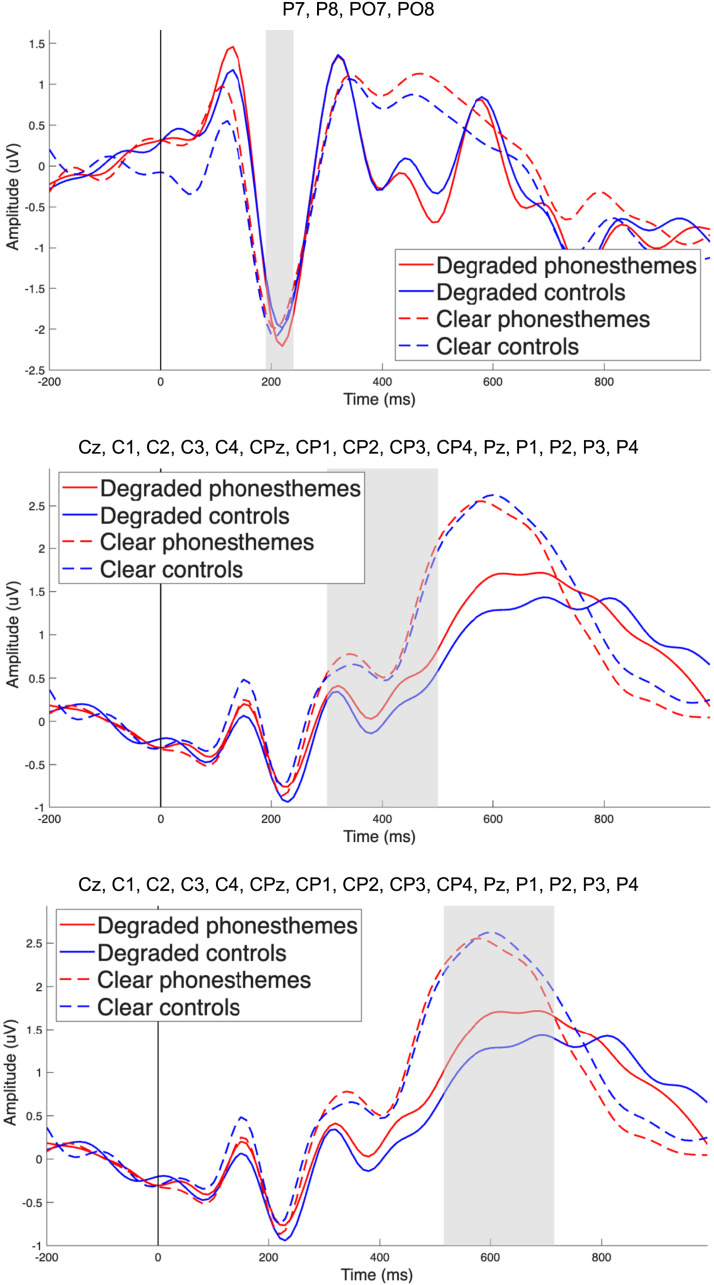
N170, N400, and P600 ERP components in the lexical decision experiment. *Note*: Grey areas indicate the time windows of interest.

As illustrated in Figure 3, negative EEG amplitude in the occipitotemporal sites peaks at 215 ms after stimulus onset. We therefore averaged amplitude across those four electrodes from 190 ms to 240 ms, within each trial. Consistent with the behavioural results, we observed a significant main effect of word type, such that phonesthemes increased N170 amplitude relative to controls, and consistent with Maurer et al. (2005), we observed a significant main effect of hemisphere, such that the N170 amplitude was greater on the left side. See Table 3. To our surprise, we did not observe a significant main effect of presentation. Instead, there were significant interactions, such that the N170 amplitude for phonesthemic words was amplified when words were clearly presented and in the left hemisphere. Given that the N170 indexes visual word recognition, these results support the hypothesis that phonesthemes facilitate visual word recognition.

#### N400 Effect.

**Figure 4.**
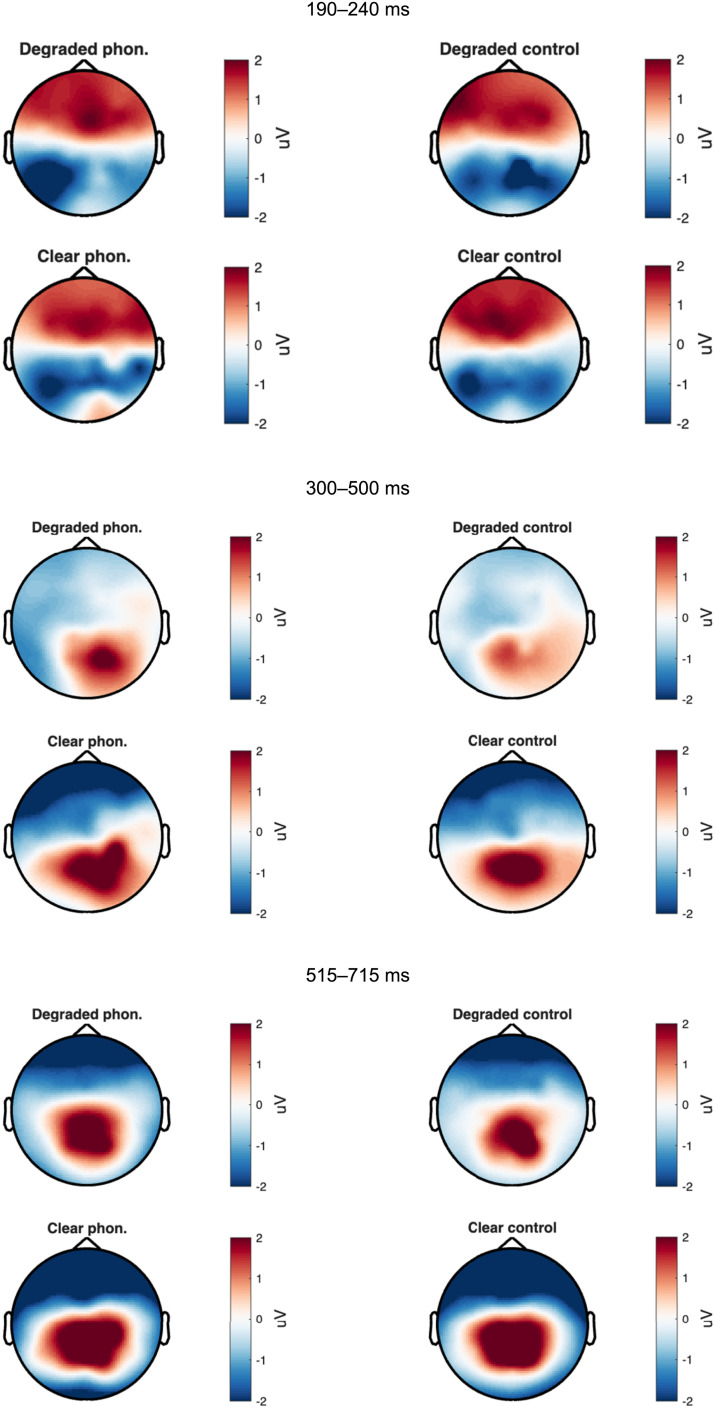
EEG amplitude during N170, N400, and P600 windows.

As reported in Table 3, we observed a significant main effect of presentation in the N400 window across the fifteen centroparietal sites, such that amplitude is more negative when words are visually degraded. Neither the main effect of word type nor its interaction with presentation approached significance. However, as illustrated in Figure 4 (and Figure 8), the negative amplitude during the N400 window is far more frontal than our analysis of centroparietal sites captures, so as a follow-up analysis, we investigated amplitude between 300 and 500 ms across the whole brain. We sum coded anterior (0.5) versus posterior sites (−0.5) and fit a three-way interaction with word type and presentation. As reported in Table 3, this analysis confirms that the frontal sites are significantly more negative during that window, and the interaction of presentation with site is significantly positive, indicating that the more negative amplitude observed in the degraded condition is more pronounced in posterior sites. No interactions with word type approach significance.

#### P600 Effect.

As illustrated in Figure 3, positive EEG amplitude in the centroparietal sites peaks at 615 ms after stimulus onset during the P600 window. We therefore averaged amplitude across those fifteen electrodes from 515 ms to 715 ms, within each trial. We observed a significant negative effect of presentation, such that the positive-going ERP is moderated in the degraded condition; a significant positive effect of word type, such that amplitude is more positive for phonesthemes that control words; and a significantly positive interaction, such that the boost for phonesthemes is greater in the degraded condition.

### Discussion

In both experiments, participants made lexical decisions faster and more accurately for words containing phonesthemes than for controls. This suggests that phonesthemes facilitate visual word recognition, which builds on findings about iconicity and form–meaning systematicity (e.g., Marelli et al., 2015; Sidhu et al., 2020) by associating this effect with quasi-morphological constituents. In the lab-based experiment, words containing phonesthemes elicited larger N170 and P600 ERPs than controls did. In both cases, the main effect of word type was in the same direction as the main effect of presentation (i.e., N170s were more negative and P600s were more positive when words contained phonesthemes and when words were clearly presented), which supports our conclusion that phonesthemes facilitate word processing.

## LEXICAL DECISION MEGASTUDIES

To corroborate the main effect of word type from Lexical Decisions About Visually Degraded Words, we investigated the impact of phonesthemes on visual word recognition in four megastudies, comprising visual and auditory lexical decisions for thousands of items by hundreds of participants.

### Materials and Methods

We used item-level RT and accuracy from the English Lexicon Project (ELP; Balota et al., 2007), British Lexicon Project (BLP; Keuleers et al., 2012), Massive Auditory Lexical Decision database (MALD; Tucker et al., 2019), and Auditory English Lexicon Project (AELP; Goh et al., 2020), gathered from the South Carolina Psycholinguistics Metabase (Gao et al., 2023). The ELP has responses from 816 participants to 40,481 words; the BLP has responses from 78 participants to 28,730 words; the MALD has responses from 231 participants for 26,793 words; and the AELP has responses from 561 participants to 10,170 words. The ELP presented targets words visually to participants from six American universities; the BLP presented words visually to participants from a British university; the MALD presented words auditorily to participants from a Canadian university; and the AELP presented words auditorily to participants from a Singaporean university.

We investigated words that are labelled as monomorphemic by MorphoLex and that have word frequency counts, concreteness ratings, iconicity ratings, and semantic neighbourhood density values. To investigate whether phonesthemes influence word recognition, we identified words that begin or end with the phonesthemes from Otis and Sagi (2008) and that share a semantic feature with any other word that begins or ends with the same phonestheme. We defined semantic features as WordNet hypernyms (Miller, 1995), available via the natural language processing toolkit (NLTK; Bird et al., 2009). For example, *crash* and *smash* end with the phonestheme /ash/ and share the hypernym “collide”, and *glow* and *gleam* start with the phonestheme /gl/ and share the hypernym “radiate”. See the Python scripts and materials on OSF.

In the ELP, 518 words are phonesthemic, out of 5,965 monomorphemic words with values for all lexical variables; in the BLP, 516 of 5,187 are phonesthemic; in the AELP, 408 of 4,435 are phonesthemic; and in the MALD, 385 of 4,766 are phonesthemic. We controlled for confounding variables in two sets of analyses. First, we regressed RT and accuracy on word frequency, bigram frequency, orthographic length, concreteness rating, iconicity rating, and semantic neighbourhood density, in linear regression models. We then regressed the residuals on the phonestheme factor, treatment coded with non-phonesthemic words as the reference level, so a negative effect of the phonestheme factor on RT would indicate faster responses, and a positive effect on accuracy would indicate a higher proportion of correct decisions. In this way, we fit eight models, with RT and accuracy for each of four databases. Note that these are item-level means, so we do not use mixed effects models.

Second, we used the MatchIt R package to match pairs of words with and without phonesthemes on the six lexical variables (Ho et al., 2011). Again, we regressed RT and accuracy on the lexical variables and then regressed the residuals on the phonestheme factor. We repeated this process a thousand times per megastudy, shuffling the order of rows at the start of each iteration to account for the imperfect nature of item matching. To identify significant effects, we used empirical *p*-values, following Davison and Hinkley (1997): We counted the number of regression models where the effect of phonesthemes is positive, for RT, or negative, for accuracy, added 1 to that value, and divided by 1,001. For example, if the phonestheme factor has a negative effect on ELP RT in 999 out of 1,000 models (i.e., if in the large majority of cases, phonesthemes speed up word recognition), the *p*-value would be .002, i.e., 2 ÷ 1001. By adding 1 to the numerator and denominator, this method avoids false positives in small samples (e.g., 10 out of 10 negative slopes would have an empirical *p*-value of .091, i.e., 1 ÷ 11, whereas 1,000 out of 1,000 negative slopes would be less than .001, i.e., 1 ÷ 1001).

### Results

**Figure 5.**
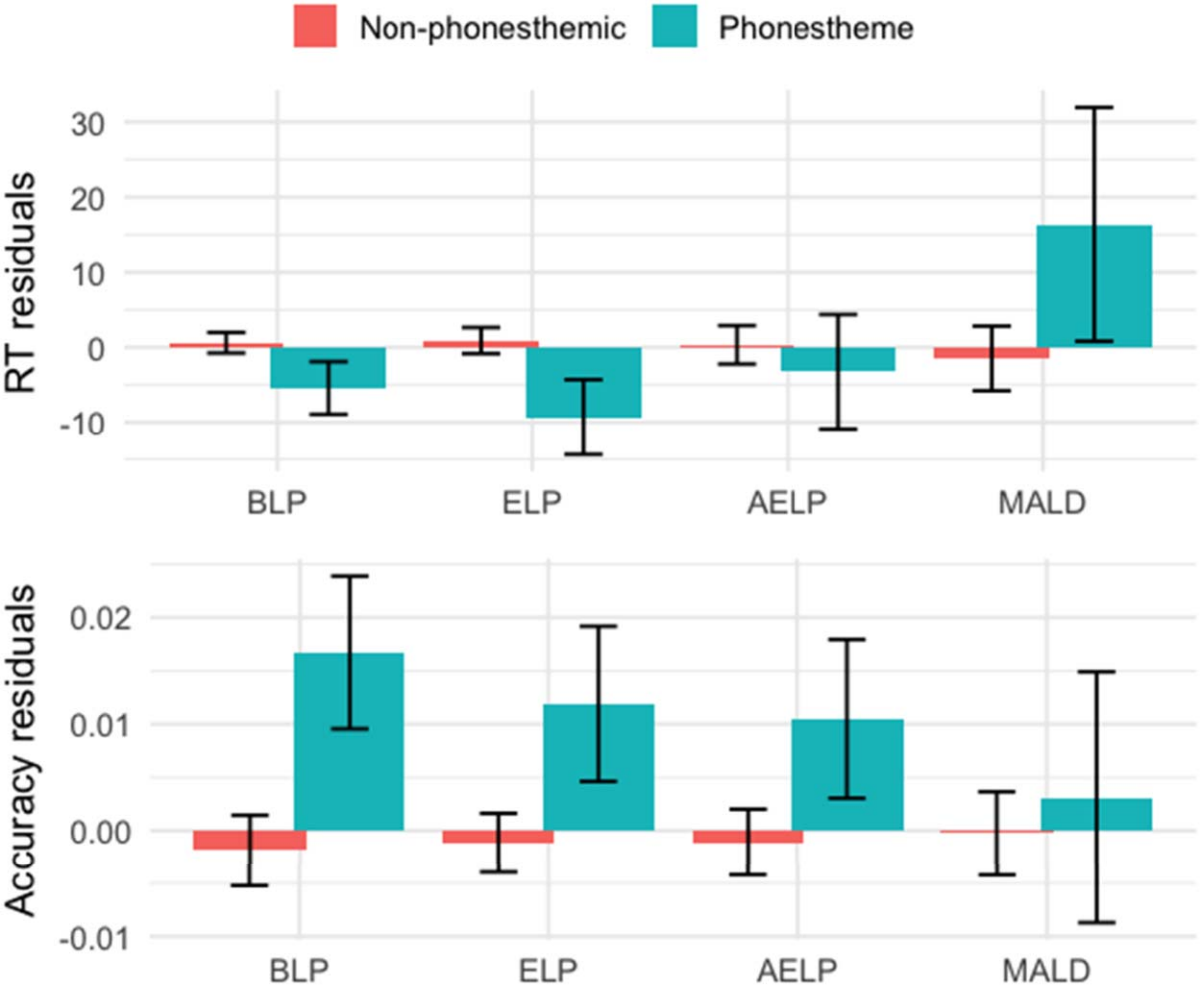
Effects of phonesthemes on RT and accuracy in four lexical decision megastudies. *Note*: Error bars indicate 95% CIs.

As reported in Tables 4 and 5 and illustrated in Figure 5, phonesthemes facilitated visual word recognition. Phonesthemes predicted significantly faster word recognition in the ELP and BLP, both in the regression models with all items and in most of the 1,000 iterations with matched items: Phonesthemes predicted faster recognition in 971 cases for the ELP (*p* = .030) and in 954 cases for the BLP (*p* = .047). Phonesthemes also predicted more accurate visual word recognition, though for ELP, the empirical *p*-value is only marginally significant: Responses to words containing phonesthemes were more accurate in 942 cases for the ELP (*p* = .059) and 985 cases for the BLP (*p* = .016). Phonesthemes improved accuracy in the AELP, but when matching items, the effect was facilitatory in only 923 of 1,000 iterations, so the empirical *p*-value does not quite reach significance (*p* = .078), and the effect on RT is far from significant. In the MALD, phonesthemes instead led to slower word recognition, but this effect held in only 853 of 1,000 iterations, which is not significant (*p* = .148).

**Table 4.** Mean (*SD*) for RT and accuracy in four lexical decision megastudies

	*N*	Mean RT (*SD*)	Mean acc. (*SD*)
ELP			
Phonesthemic	518	668 (80)	92.2 (10.4)
Non-phonesthemic	5,447	691 (93)	90.4 (12.5)
BLP			
Phonesthemic	516	589 (56)	93.5 (9.8)
Non-phonesthemic	4,671	598 (66)	90.9 (13.7)
AELP			
Phonesthemic	408	931 (88)	91.4 (8.6)
Non-phonesthemic	4,027	940 (84)	90.1 (11.0)
MALD			
Phonesthemic	385	894 (164)	94.2 (12.5)
Non-phonesthemic	4,381	884 (150)	93.7 (13.7)

**Table 5.** Results of linear regression models for four lexical decision megastudies

	*B*	*SE*	*p*	Mean *B*	N neg. / pos.	Empirical *p*
ELP						
RT	−10.68	2.98	.0003	−10.70	971	.030
Accuracy	.012	.005	.009	.013	942	.059
BLP						
RT	−6.70	2.19	.002	−6.81	954	.047
Accuracy	.019	.005	.0003	.019	985	.016
AELP						
RT	−4.01	4.29	.452	−4.04	683	.318
Accuracy	.013	.005	.013	.012	923	.078
MALD						
RT	18.15	7.77	.020	18.40	147	.853
Accuracy	.004	.007	.567	.004	637	.364

*Note*. These models regress residuals on whether words contain phonesthemes (after regressing item-level RT/accuracy on word frequency, orthographic length, bigram frequency, concreteness rating, iconicity rating, and semantic neighbourhood density). The predictor is treatment coded, so positive effects correspond to slower RT or higher accuracy. The left side reports coefficients, standard errors, and *p*-values for eight regression models. The right side reports results from 8,000 regression models (1,000 sets of paired items matched on the six lexical variables): mean coefficients, the number of facilitatory coefficients, and empirical *p*-values.

The discrepancy between visual and auditory word recognition supports Tucker et al. (2019), who motivated the MALD by cautioning against the tendency to generalize effects found in visual word processing. Tucker et al. contrast the gradual integration of auditory word recognition with the fixation on chunks in visual word recognition, which could explain why chunk-like phonesthemes seem more salient during visual processing. Furthermore, some phonesthemes are defined orthographically, such as /wr/ in *wrangle* and *wrest*. This emphasis on orthography is consistent with morphology experiments that rely on masked priming (e.g., Rastle et al., 2004), and it raises important questions about how the effects of systematicity and compositionality vary by modality (cf. Kouider & Dupoux, 2005; Ussishkin et al., 2015). Finally, note that responses are far slower in auditory decisions (911 ms on average, compared to 646 ms for the ELP and BLP), consistent with fundamental differences in word recognition that change the effect of phonesthemes.

## SEMANTIC RELATEDNESS DECISIONS ABOUT MISLEADING WORD FORMS

To investigate whether phonesthemes influence word meaning access, we conducted two semantic relatedness experiments, using words that contain misleading phonesthemes, i.e., which lack the meaning typically associated with that phonestheme. For example, unlike many other /gl/ words, *glove* does not refer to light. The first experiment was conducted online, and the second was conducted in a lab while measuring EEG amplitude. These experiments have a one-factor, two-level design (word type: misleading or control, manipulated within participants and within items). All data and scripts are available at osf.io/mhv4b.

### Methods

#### Participants.

For the online experiment, we recruited 64 native English speakers from Prolific (43 female, 21 male, mean age = 35.1, range = 19–50). They were paid £1 for a 5-minute task. For the lab-based experiment, we used the same 36 participants in the same two-hour session as in the lexical decision experiment, with the same EEG recording and preprocessing.

#### Stimuli and Procedure.

For the online experiment, we designed 126 items in two conditions. Each item comprises a cue word that is related to the meaning of a phonestheme but does not contain that phonestheme (e.g., *shine* is related to /gl/) followed by a target word. In the misleading condition, the target contains the phonestheme that is related to the meaning of the cue, but the target does not convey that meaning (e.g., *glove* contains /gl/). Therefore, the cues and targets are not semantically related. In the control condition, the target has a similar meaning to the misleading target but lacks the phonestheme (e.g., *mitten*). We also designed 126 semantically related cue–filler pairs. To keep the online experiment short, we split the items into three groups of 42, for a total of six lists (manipulating the target type within each item), with 42 fillers per list. For the lab-based experiment, each participant saw all items, so we reduced the stimuli to 100 items and 100 fillers.

Table 1 (p. 6) compares controls and misleading items on the same six lexical variables as in Lexical Decisions About Visually Degraded Words and Lexical Decision Megastudies: orthographic length, word frequency, bigram frequency, semantic neighbourhood density, concreteness rating, and iconicity rating. It additionally compares the semantic and orthographic similarity of cue–target pairs. Semantic similarity is quantified as the cosine similarity of word2vec embeddings. These embeddings are the product of a neural network that learns the linguistic contexts where words occur in the Google news corpora, represented in a 300-dimension vector space (Mikolov et al., 2013). Orthographic similarity is quantified as edit distance (i.e., the number of letters that must be changed to make the cue and target forms identical), normalized for length (i.e., divided by the maximum possible edit distance), and inverted to convey similarity rather than distance. For example, *cat* and *catch* have a similarity score of 0.4: the raw distance, 3, divided by the maximum distance, 5, subtracted from 1. In paired *t-*tests comparing misleading and control conditions, *p*-values are less than .2 for semantic neighbourhood density in the lab-based experiment (*p* = .015); for bigram frequency in the lab-based experiment (*p* = .026) and the online experiment (*p* < .001); for orthographic similarity in the lab-based experiment (*p* = .185) and the online experiment (*p* = .126); and for iconicity in the lab-based experiment (*p* = .028) and the online experiment (*p* = .002). To control for such differences between conditions, we regressed RT, accuracy, and EEG amplitude on these eight predictors and then used the residuals as our dependent variable, as in the lexical decision experiment.

#### Procedure.

**Figure 6.**
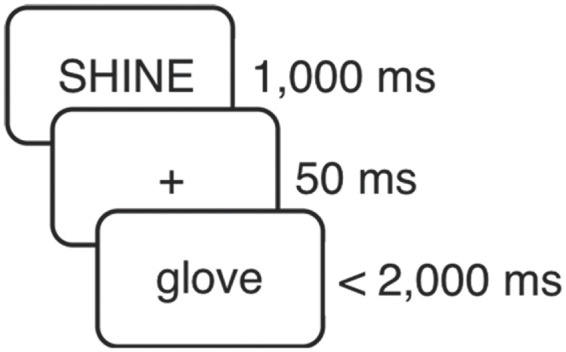
Example item from the semantic relatedness experiment.

The online experiment was implemented on Gorilla.sc (Anwyl-Irvine et al., 2020). The based-based experiment was implemented on OpenSesame (Mathôt et al., 2012). For each experiment, a cue word was presented for 1,000 ms, followed by a fixation cross for 200 ms, and then the target word for up to 2,000 ms. See Figure 6. Participants indicated whether the cue–target pair was semantically related by pressing A or L; the key which indicated related or not was counterbalanced across participants.

#### Data Analysis.

For both semantic relatedness experiments, we measured RT and accuracy (i.e., “no” decisions for target items, or “yes” decisions for related fillers). We regressed RT and accuracy on the eight lexical variables described above, and we used the residuals as our dependent variables. As with the lexical decision experiments, we excluded participants who responded correctly on less than two-thirds of trials, and for RT analyses, we included only correct trials and excluded trials faster than 200 ms or more than 2.5 *SD* above the mean RT by subject. We again conducted mixed effects modelling with the maximal random effects structure justified by the data, sum coding word type (control = −0.5, misleading = 0.5, then mean-centred). For the lab-based experiment, we investigated N170, N400, and P600 ERP components, in the same way as in the lexical decision experiment.

### Behavioural Result

**Figure 7.**
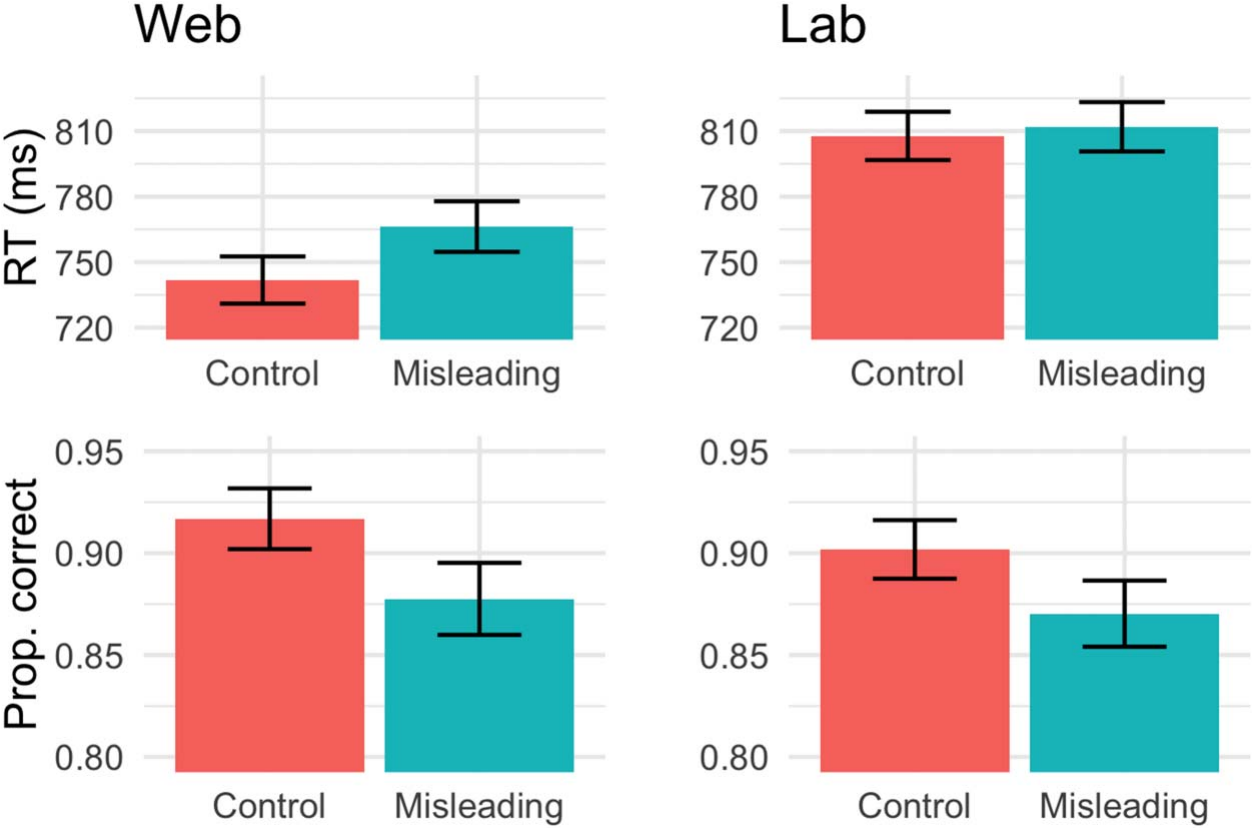
Response time and accuracy in the semantic relatedness experiments.

For the online experiment, we excluded one out of 64 participants for low accuracy, and we excluded 60 of 2,374 remaining trials (2.5%) for outlying RT values. For the lab-based experiment, we excluded three out of 36 participants for low accuracy, and we excluded 78 of 2,924 remaining trials (2.7%) for outlying RT values. As shown in Tables 6 and 7 and Figure 7, we observed a significant effect of target type on both RT and accuracy in the online experiment: Participants were slower and less likely to decide that a cue–target pair was unrelated when the target misleadingly contained a phonestheme. In the lab-based experiment, this increased tendency to decide that misleading words were related to cues was numerically similar to the online experiment but did not reach significance. Furthermore, the effect of target type on RT was far from significant. Participants were slower in the lab-based experiment than online (810 ms compared to 767 ms), which could explain the null effect of misleading phonesthemes on RT, as we unpack in Differences in the Online and Lab-Based Results. Overall, these behavioural effects provide limited support our hypothesis that misleading phonesthemes, such as /gl/ in *glove*, impede word meaning access.

**Table 6.** Mean (*SD*) response time and accuracy for semantic relatedness decisions

	RT (ms)		Accuracy	
	Web	Lab	Web	Lab
Control	742 (189)	808 (214)	.92 (.28)	.90 (.30)
Misleading	766 (198)	812 (216)	.88 (.33)	.87 (.34)

**Table 7.** Results of mixed effects regression models for semantic relatedness decisions

	Web-based RT			Lab-based RT		
	*B*	*SE*	*p*	*B*	*SE*	*p*
Target type	22.9	7.1	.0016	5.59	7.9	.4830
	Web-based accuracy			Lab-based accuracy		
	*B*	*SE*	*p*	*B*	*SE*	*p*
Target type	−0.08	0.04	.0282	−0.07	0.04	.0811
				N170 ERP		
				*B*	*SE*	*p*
Target type				0.16	0.23	.4930
Hemisphere				−0.38	0.41	.3650
Target type: Hemisphere				0.29	0.47	.5420
				N400 ERP		
				*B*	*SE*	*p*
Target type				−0.006	0.11	.9570
				P600 ERP		
				*B*	*SE*	*p*
Target type				0.08	0.12	.4880
				N400, whole brain		
				*B*	*SE*	*p*
Target type				−0.21	0.10	.0403
Anteriority				−1.56	0.27	2e−6
Target type: Anteriority				−0.39	0.20	.0588

*Note*. The dependent variables are the residuals of RT/accuracy/ERP amplitude after being regressed on eight lexical variables: word2vec cosine similarity, orthographic similarity, orthographic length, word frequency, bigram frequency, concreteness rating, iconicity rating, and semantic neighbourhood density. All predictors are sum coded and mean centred, with misleading phonesthemes and left/anterior sites as the positive values.

### ERP Results

#### N170 Effect.

**Figure 8.**
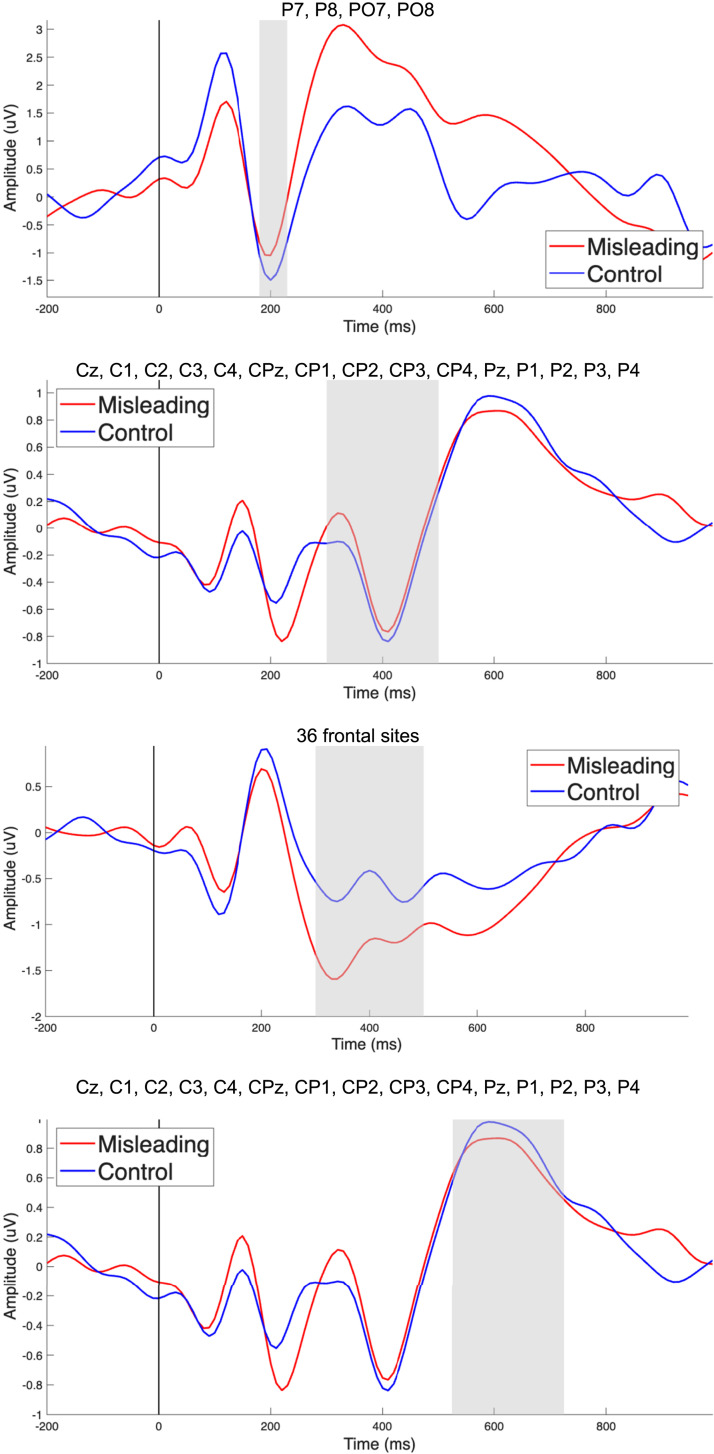
N170, N400, and P600 ERP components in the semantic relatedness experiment. *Note*: Grey areas indicate the time windows of interest.

**Figure 9.**
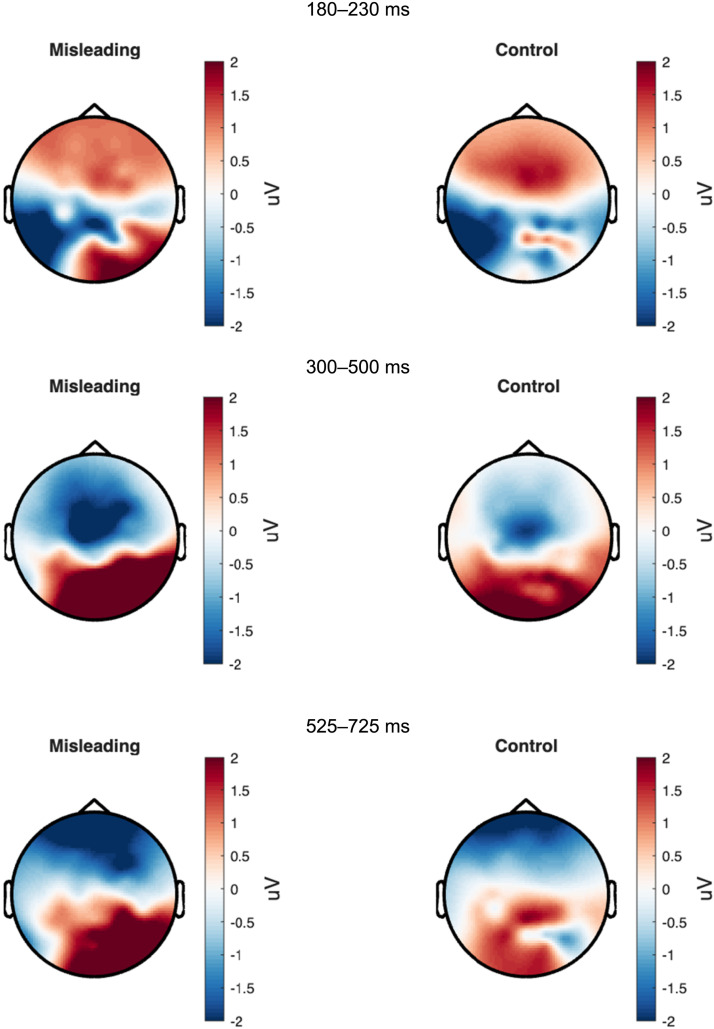
EEG Amplitude during the N170, N400, and P600 windows.

As illustrated in Figure 8, negative EEG amplitude in the occipitotemporal sites peaks at 205 ms after stimulus onset. We therefore averaged amplitude across those four electrodes from 180 ms to 230 ms, within each trial. The difference in N170 effect between the control and misleading conditions is far from significant, providing no evidence that misleading phonesthemes impeded visual word recognition during this semantic relatedness experiment. As in the lexical decision experiment and as illustrated in Figure 9, N170 amplitude is numerically more negative in the left hemisphere. However, the hemisphere factor is far from significant, as is its interaction with the word type factor.

#### N400 Effect.

As with the N170 effect, the difference between the misleading and control conditions is far from significant in the N400 sites from 300–500 ms. However, as in the lexical decision experiment, the amplitude is more frontal than we had anticipated. In a follow-up whole-brain analysis, reported in Table 7, the main effect of word type is significant, such that misleading phonesthemes elicit larger N400 effects than controls. This provides some evidence that misleading phonesthemes impede word meaning access, but it is important to note that our planned analysis of N400 amplitude in centroparietal sites results in a null effect.

#### P600 Effect.

As reported in Table 7 and illustrated in Figure 8, the effect of word type is far from significant in the P600 window and sites. The results therefore provide no evidence that words containing misleading phonesthemes elicit larger P600s than controls do, contrary to the hypothesis that quasi-morphological constituents which cannot be easily integrated into incongruent word meanings increase processing costs.

### Differences in the Online and Lab-Based Results

In the online semantic relatedness experiment, the difference in accuracy between the misleading and control conditions was small but significant: 3.9% higher accuracy in the control condition. In the lab-based experiment, the difference failed to reach significance but was only slightly smaller than online: 3.1% higher accuracy in the control condition. As we report in full on OSF, this difference is negligible: When pooling the data and regressing accuracy on word type and experiment, their interaction is far from significant (*p* = .493). The lack of a significant effect of word type in the lab-based experiment suggests that it was statistically underpowered to detect subtle shifts in interpretation.

**Figure 10.**
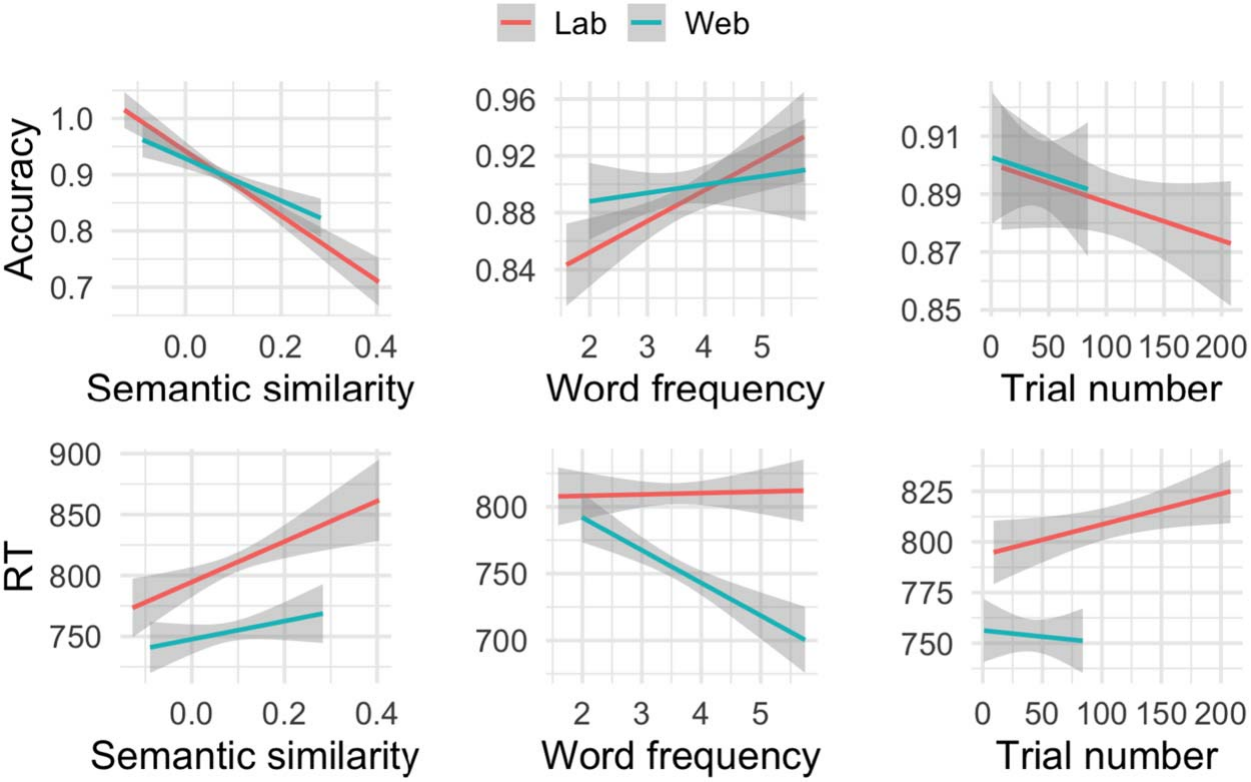
Semantic relatedness decisions online and in the lab. *Note*: Shaded areas indicate 95% CIs, based on binary logistic regression (accuracy) or linear regression (RT). Recall that this experiment involves decisions about semantically unrelated words (e.g., *shine* and *glove*), hence the decrease in accuracy (i.e., fewer “no” decisions) as cue–target pairs increase in semantic similarity (i.e., people are more likely to decide semantically similar pairs are related and are slower to decide they are not related).

The difference in RT across experiments is harder to explain. When pooling data and regressing RT on word type and experiment, the main effect of experiment is highly significant (*p* < .0001), whereas the interaction is only marginally significant (*p* = .078). As is apparent in Figure 7, participants were far slower in the lab-based experiment (810 ms on average, compared to 754 ms). To investigate this difference, we regressed RT on experiment and its interaction with each of the eight lexical variables described in Table 1. As illustrated in Figure 10 and reported in full on OSF, word frequency is the only lexical variable that has a significant interaction with experiment (*p* = .021), and it is a large interaction (*B* = 16.9), cancelling out the main effect of word frequency (*B* = −12.1): People made faster decisions about more common targets in the online experiment but not in the lab-based experiment. In contrast, when regressing accuracy on the interaction of experiment with each lexical variable, none of the interactions approach significance.

So, in the lab-based experiment, accuracy decreased only marginally, yet responses slowed down substantially, and the influence of word frequency disappeared. This implies that participants adopted a different strategy which focused less on speed. Perhaps in-person participants who committed to a two-hour session felt less pressure to get the task over with than participants who signed up for a five-minute study online. However, in the lexical decision experiment, the same group of in-person participants responded almost as quickly as online participants. The more likely explanation is fatigue: The semantic relatedness experiment was the last of three experiments conducted over the two-hour session, whereas the lexical decision experiment was the first. As we illustrate in Figure 10 and report in full on OSF, RT increased and accuracy decreased over the course of the semantic relatedness experiments, and more so in the lab than online. However, with few trials per participant in the online experiment, the interaction of trial number with experiment does not reach significance (*p* = .08).

## GENERAL DISCUSSION

In four experiments and in reanalyses of four megastudies, we have provided clear evidence that, in English, phonesthemes facilitate visual word recognition and limited evidence that phonesthemes affect interpretation. In two lexical decision experiments and two lexical decision megastudies, people recognized words containing phonesthemes faster and more accurately than controls, and words containing phonesthemes elicited larger N170 and P600 ERPs, especially when visually degraded. The enhanced N170 effect is consistent with the behavioural effects, such that phonesthemes facilitated recognition, but the P600 effect is less straightforward. We had hypothesized that treating phonesthemes like quasi-morphological constituents would increase integration costs (i.e., phonesthemes may increase morphosyntactic complexity). However, the most pronounced effect in the P600 window was greater amplitude for clearly presented words, which are easier to process, not harder. The simpler explanation, then, is that greater P600 amplitude for phonesthemes indicates easier processing, as with RT, accuracy, and N170 amplitude. However, because this effect runs counter to our prediction, we present it as suggestive evidence and a jumping off point for future work.

In the online semantic relatedness experiment, participants were more likely to decide that, for example, the cue *shine* was related to the misleading target *glove* than to the control *mitten*, and when participants rejected the relatedness of *shine* to *glove*, they did so more slowly. This suggests that phonesthemes guide word meaning access and, consequently, that misleading phonesthemes, as in *glove*, interfere with word meaning access. However, the effect of misleading phonesthemes on RT disappeared in the lab-based experiment, seemingly because fatigued participants were less inclined to rush their decisions. Accuracy was comparable to what we observed online, though it did not reach significance, and misleading phonesthemes led to larger frontal N400 effects than controls, though the hypothesized N400 and P600 effects in the centroparietal region were far from significant.

These findings are valuable because phonesthemes complicate compositional models of morphology, according to which visual word processing involves automatically decomposing words to morphemes. Such models often invoke masked priming studies, where primes speed up lexical decisions about targets that share morphemes more than semantic relatedness can explain (e.g., Rastle et al., 2000). However, Bergen (2004) provides evidence that pairs which share phonesthemes exhibit comparable boosts in priming, despite those items defying canonical decomposition. Baayen et al. (2011, p. 467) conclude that morphology “can begin to account for these kinds of phenomena only by freeing itself from the chains of the morpheme.” To better explain such effects, distributed theories of morphology propose that “morphological sensitivity arises from… statistical regularities in form-to-meaning mapping, without the need for explicit morpheme representations” (Stevens & Plaut, 2022; p. 1673). That is, apparent constituents, such as phonesthemes, emerge as a byproduct of form–meaning systematicity. By providing evidence that form–meaning systematicity facilitates word processing independently of morphological composition, our experiments lend support to distributed theories of morphology, and they do so while building on seminal work by Bergen (2004) and correcting for an overreliance on pseudowords in the phonestheme literature. Bear in mind, though, that our experiments differ substantially from Bergen (2004) because we did not use priming. In the lexical decision experiments, we presented single target words, and in the semantic relatedness experiments, cue words were presented overtly, not subliminally or around the threshold of conscious perception, and cues and targets did not share phonesthemes in either condition (e.g., *shine* to *glove* or *mitten*). Unlike in Bergen (2004), the impact of misleading phonesthemes on semantic relatedness decisions reflects how people interpret words that contain phonesthemes, which may involve conscious associations rather than automatic decomposition.

Our findings also support the theory that form–meaning systematicity plays a role in language evolution. Kirby et al. (2022) provide evidence from toy sign languages that people have an initial preference for holistic forms when they learn individual words but that learning a vocabulary leads people to decompose those forms into serial constituents. For example, an initially holistic sign that moved downward while spiralling was segmented into a spiral followed by a downward motion. This is consistent with Smith (2014, 2016), who provides corpus evidence that phonesthemes are overrepresented in blend words (i.e., people recombine sub-morphemic strings which recur in semantically related words) and that people reanalyse familiar forms that contain phonesthemes (e.g., people interpret *flourish* in light of *flutter* and *flicker*). It is also consistent with Zingler (2017), who provides corpus evidence that phonesthemes influence word formation in English. Our experiments capture this reanalysis of holistic forms in contemporary English. For phonesthemes to facilitate visual word recognition and motivate semantic change is consistent with earlier work in form–meaning systematicity (e.g., Haslett & Cai, 2022, 2023; Marelli et al., 2015, Marelli & Amenta, 2018), but by focusing on sub-morphemic constituents, we bridge the gap between word processing and the evolution of compositionality.

Finally, as psychologically real shades of grey between morphemes and meaningless strings of letters, phonesthemes can inform methods in natural language processing. Large language models (LLMs), such as GPT-5 (OpenAI, 2025) and DeepSeek-R1 (Guo et al., 2025), segment text into chunks called tokens. Common English words are usually single tokens, but most other words are represented as combinations of subword tokens. For example, GPT-5 represents *neologism* with three tokens: ne + olog + ism. As *neologism* illustrates, tokens are not determined by morphology or semantics, so tokenization obscures meaningful constituents such as *neo* (Bostrom & Durrett, 2020; Church, 2020). Such morphologically malformed tokens can misalign representations of word meanings in humans and LLMs (e.g., Batsuren et al., 2024; Hofmann et al., 2021). However, morpheme-centric tokenization misses crucial semantic information, such as sub-morphemic category markers and phonesthemes (Gutierrez-Vasques et al., 2023; Haslett, 2025; Haslett & Cai, 2025). For example, according to GPT-5, *glimmer* and *glisten* share the token “gl”, but *glow* is a single token, and *glittering* decomposes morphologically (i.e., “glitter” + “ing”), which obscures that phonestheme from GPT-5 in its most common and representative contexts. Dynamic tokenization, or a smaller vocabulary of tokens that decomposes more words, could better convey such patterns in form–meaning mapping (e.g., Pagnoni et al., 2025). By providing evidence that phonesthemes affect how people process English words, we hope to discourage methods that hew too close to compositional theories of morphology.

## CONCLUSION

This study provides evidence from four experiments and four megastudies that phonesthemes facilitate word recognition and affect interpretation in English. Our findings support distributed theories of morphology, which view morpheme-like constituents as byproducts of form–meaning systematicity, and they support the claim that form–meaning systematicity plays a role in the cultural evolution of language, including the emergence of compositionality. By accounting for these shades of grey between morphemes and meaningless strings of letters, natural language processing methods could better align representations in large language models with human word meanings.

## ACKNOWLEDGMENT

Many thanks to Bodo Winter, Philip Thierfelder, and two anonymous reviewers for helpful comments on earlier drafts.

## FUNDING INFORMATION

This project received funding from the CUHK–University of Manchester Research Fund (Seed-corn Fund), for BY and ZGC, and the Hong Kong Research Grant Council (PhD Fellowship), for DAH.

## AUTHOR CONTRIBUTIONS

D.A.H: Conceptualization; Formal analysis; Investigation; Writing – original draft. B.Y.: Formal analysis; Funding acquisition; Investigation; Writing – review & editing. X.D.: Formal analysis; Writing – review & editing. Z.G.C.: Funding acquisition; Writing – review & editing.

## DATA AVAILABILITY STATEMENT

Materials and scripts are available on the Open Science Framework: osf.io/mhv4b.
